# Updates in the Management of Short Bowel Syndrome Secondary to Intestinal Resection in the Pediatric Population

**DOI:** 10.3390/jcm15187291

**Published:** 2026-09-19

**Authors:** Andrada-Lorena Gafton-Oghină, Elena Cernat, Elena Cojocaru, Solange Tamara Roșu, Laura Mihaela Trandafir, Jana Bernic, Viorel Țarcă, Alina Costina Luca, Dana Elena Mîndru, Elena Țarcă

**Affiliations:** 1 Grigore T. Popa University of Medicine and Pharmacy, 700115 Iasi, Romania; andrada-lorena.gafton-oghina@umfiasi.ro (A.-L.G.-O.); elena.cernat@d.umfiasi.ro (E.C.); elena2.cojocaru@umfiasi.ro (E.C.); laura.trandafir@umfiasi.ro (L.M.T.); acluca@yahoo.com (A.C.L.); mindru.dana@umfiasi.ro (D.E.M.); tarca.elena@umfiasi.ro (E.Ț.); 2 Leeds Teaching Hospitals NHS Trust, Leeds LS1 3EX, UK; 3 “Nicolae Testemițanu” State University of Medicine and Pharmacy, MD-2001 Chisinau, Moldova; jana.bernic@usmf.md; 4 Faculty of Medicine, Apollonia University, 700511 Iasi, Romania; viorel.tarca@univapollonia.ro

**Keywords:** intestinal resection, short-bowel syndrome, pediatric population, parenteral nutrition, intestinal failure

## Abstract

**Background:** Short bowel syndrome (SBS) secondary to intestinal resection is the most common cause of intestinal failure (IF) in children and is associated with prolonged parenteral support (PS), impaired growth, and substantial morbidity. SBS outcomes have improved dramatically over the past ten years due to advancements in intestinal rehabilitation programs, nutritional approaches, pharmaceutical treatments, and surgical restoration. **Objective:** With an emphasis on surgical decision-making, intestinal rehabilitation, nutritional optimization, pharmacologic advancements and predictors of enteral autonomy, the goal is to thoroughly review and synthesize the most recent data on managing pediatric SBS after intestinal resection. **Methods:** This systematic review was conducted in accordance with the PRISMA 2020 statement using PubMed and Embase. Eligible primary clinical studies evaluated management strategies or clinically relevant outcomes in pediatric patients with SBS secondary to intestinal resection. Secondary evidence sources, including review articles and evidence-based position papers, were considered separately and were used to contextualize the primary findings and contemporary clinical recommendations. The methodological quality and risk of bias of primary studies were assessed using design-appropriate critical appraisal tools. Owing to substantial clinical and methodological heterogeneity, the evidence was synthesized qualitatively. **Results:** Multidisciplinary intestinal rehabilitation programs have become the mainstay of therapy, increasing the rates of enteral autonomy and improving survival. While optimal PS administration reduces intestinal failure–associated liver disease (IFALD) and catheter-related bloodstream infections (CRBSIs), early, organized enteral feeding encourages intestinal adaptation. Significant advances in decreasing PS requirements have been achieved by pharmacological therapies such as glucagon-like peptide-2 (GLP-2) analogs. Advances in multidisciplinary intestinal rehabilitation have reduced the need for intestinal transplantation and have refined patient selection for autologous bowel reconstruction. Consequently, procedures such as Serial Transverse Enteroplasty (STEP) and Longitudinal Intestinal Lengthening and Tailoring (LILT) are now reserved for highly selected patients with persistent bowel dilatation, dysmotility, and inadequate intestinal adaptation despite optimized nutritional and medical therapy. **Conclusions:** Following intestinal resection, pediatric SBS is now managed through multidisciplinary, protocol-driven intestinal rehabilitation. Outcomes have improved through the integration of nutritional, pharmacological, and surgical strategies tailored to each patient’s residual anatomy and adaptive potential. Future prospective pediatric studies are needed to standardize outcome measures and clarify the optimal timing and sequencing of nutritional, pharmacological, and surgical interventions during intestinal rehabilitation.

## 1. Introduction

Short Bowel Syndrome (SBS) is the primary cause of intestinal failure (IF) in children and is characterized by a state of intestinal insufficiency secondary to a significant loss of functional small intestine [1,2]. In children, SBS typically occurs after surgical resection for conditions that predominantly affect neonates and infants, such as necrotizing enterocolitis, intestinal atresia, gastroschisis, midgut volvulus, or ischemic injury [2,3]. Although survival has increased due to advancements in pediatric surgery and neonatal intensive care, a large percentage of these children still require prolonged parenteral support (PS), which is linked to considerable morbidity and healthcare costs [4,5].

The clinical outcomes of pediatric SBS are quite heterogeneous and depend on a complex interplay of physiological and anatomical parameters, such as the residual small-bowel length, colon-in-continuity, ileocecal valve (ICV) presence, and bowel quality [6,7,8]. The most severe phenotypes are neonatal-onset SBS and ultrashort bowel syndrome, which have historically been linked to high mortality and early intestinal transplant referrals [5]. However, even in the context of markedly reduced residual bowel length, recent cohort data demonstrate improved long-term survival and the possibility of achieving enteral autonomy in selected patients [5,6].

Over the last decade, management paradigms have shifted from isolated supportive care toward structured intestinal rehabilitation programs (IRPs), which integrate pediatric surgery, nutrition/gastroenterology, dieticians, pharmacy, nursing, SALT and psychosocial support within a coordinated care model [9,10]. Registry-based and cohort studies have demonstrated that IRPs are associated with improved survival, reduced rates of intestinal failure–associated liver disease (IFALD) and catheter-related bloodstream infections (CRBSIs), and higher likelihood of achieving enteral autonomy [9,10].

Concurrent advances have been made in surgical decision-making, while nutritional strategies emphasizing early and gradual enteral feeding have been shown to promote intestinal adaptation and reduce dependence on PS [3,11,12]. Better monitoring of PS-related comorbidities, such as vitamin deficiencies and metabolic bone disease, has improved long-term outcomes [13,14]. Predictive models and disease severity score systems have enabled early risk classification for enteral autonomy and informed surgical intervention scheduling [15,16,17,18,19,20].

The aim of this systematic review was to synthesize contemporary primary clinical evidence on advances in the management of pediatric short bowel syndrome following intestinal resection, with particular emphasis on surgical decision-making, multidisciplinary intestinal rehabilitation, and factors influencing long-term outcomes. Secondary evidence and contemporary position statements were additionally considered to contextualize the primary findings and delineate current clinical recommendations.

To ensure methodological transparency, the review protocol was prospectively registered in the International Prospective Register of Systematic Reviews (PROSPERO; CRD420251032911).

## 2. Materials and Methods

**Figure 1:**
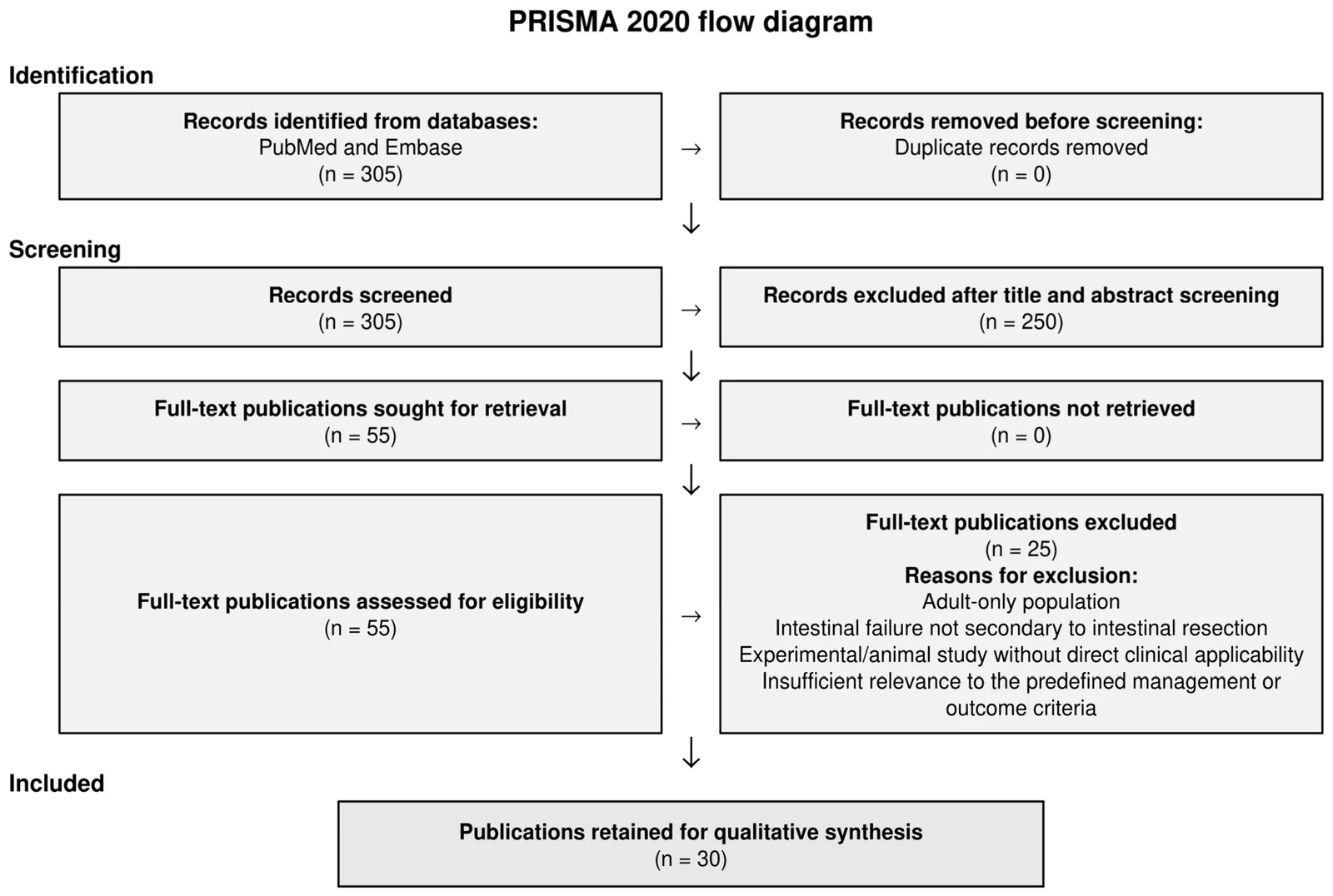
PRISMA 2020 flow diagram of the literature search and publication-selection process [21].

This systematic review was conducted in accordance with the Preferred Reporting Items for Systematic Reviews and Meta-Analyses (PRISMA) 2020 guidelines. The literature search and study selection are summarized in Figure 1. The flow diagram illustrates the number of records identified, screened, assessed for eligibility, and ultimately included in the review after application of the predefined inclusion and exclusion criteria.

### 2.1. Eligibility Criteria

Studies were considered eligible for inclusion if they fulfilled all of the following criteria:

1. Included a pediatric population (patients younger than 18 years of age) diagnosed with short bowel syndrome or intestinal failure secondary to intestinal resection.
2. Evaluated one or more aspects of multidisciplinary management, including intestinal rehabilitation programs, enteral or parenteral nutrition, pharmacological therapies (including glucagon-like peptide-2 [GLP-2] analogs), surgical rehabilitation techniques such as Serial Transverse Enteroplasty (STEP) and Longitudinal Intestinal Lengthening and Tailoring (LILT)/Bianchi, or intestinal transplantation.
3. Reported clinically relevant outcomes, including achievement of enteral autonomy, reduction in parenteral support requirements, growth and nutritional status, parenteral nutrition (PN)-related complications, intestinal adaptation and patient survival [4,9,10,18,22].

For the primary evidence synthesis, eligible publications comprised original clinical studies reporting patient- or clinician-level data, including prospective and retrospective observational studies, registry-based analyses, clinical case series, interventional studies, and survey-based studies, provided that they fulfilled the predefined population, management, and outcome criteria. Secondary evidence sources, including narrative, systematic, or integrative reviews and evidence-based position papers, were identified within the same literature search but were considered separately from primary studies. These publications were retained to contextualize the primary evidence, summarize contemporary concepts and clinical recommendations, and facilitate supplementary reference screening; they were not treated as independent sources of primary outcome data.

Publications were excluded if they focused exclusively on adult populations, investigated intestinal failure unrelated to intestinal resection, consisted solely of experimental or animal studies without direct clinical applicability, or did not provide clinically relevant information regarding the management or outcomes of pediatric SBS. Publications without original patient- or clinician-level data were not included in the primary evidence synthesis or formal risk-of-bias assessment but could be retained as secondary contextual evidence when directly relevant to the scope of the review [1,23].

### 2.2. Information Sources and Search Strategy

A systematic literature search was conducted in PubMed and Embase to identify contemporary evidence on the management of pediatric short bowel syndrome (SBS) secondary to intestinal resection. The initial literature search was performed in April 2025 and was subsequently updated on 10 May 2026 to identify potentially eligible publications available before completion of the review. The search covered publications from January 2015 through 10 May 2026. The search was restricted to articles published in English.

The search strategy combined controlled vocabulary, including Medical Subject Headings (MeSH) in PubMed and Emtree terms in Embase, with corresponding free-text terms. The search was structured around three principal concepts: (1) short bowel syndrome and intestinal failure; (2) the pediatric population; and (3) therapeutic management and clinically relevant outcomes. Search terms included “short bowel syndrome,” “intestinal failure,” “pediatric,” “child,” “infant,” “intestinal resection,” “intestinal rehabilitation,” “enteral autonomy,” “parenteral nutrition,” “teduglutide,” “GLP-2,” and “intestinal transplantation.” Synonymous terms within each concept were combined using the Boolean operator OR, and the principal concepts were combined using AND. Database-specific controlled vocabulary and syntax were adapted as appropriate for PubMed and Embase [24,25].

To maximize retrieval of relevant evidence, the reference lists of eligible primary studies and relevant review articles and position papers were additionally screened for potentially eligible publications not identified through the electronic database searches.

### 2.3. Study Selection and Data Extraction

All records retrieved through the electronic database searches were assessed for duplication before screening. No duplicate records were identified; consequently, all 305 retrieved records proceeded to title and abstract screening. Titles and abstracts were independently screened by two reviewers according to the predefined eligibility criteria. Publications considered potentially eligible underwent full-text assessment, which was independently performed by the same two reviewers. Disagreements regarding study eligibility were resolved through discussion and consensus; when consensus could not be reached, a third reviewer was consulted.

Data extraction was performed by one reviewer using a predefined set of variables and was independently verified for accuracy and completeness by a second reviewer. Extracted information included study design, year of publication, patient population, underlying etiology of SBS, residual bowel anatomy, characteristics of intestinal rehabilitation, nutritional and pharmacological interventions, surgical procedures, reported clinical outcomes, and duration of follow-up. Any discrepancies identified during verification were resolved through discussion and re-examination of the original publication [9,15,16].

### 2.4. Synthesis

Because of the marked heterogeneity among the included studies with respect to study design, patient populations, residual bowel anatomy, therapeutic strategies, duration of follow-up, and reported outcome measures, a quantitative meta-analysis was not considered appropriate. Instead, the available evidence was synthesized using a narrative approach. The qualitative synthesis was structured according to the type and evidentiary role of the included publications. Primary clinical studies constituted the principal evidence base for statements concerning clinical outcomes, therapeutic effects, predictors of enteral autonomy, and associations between anatomical or clinical characteristics and outcome. Secondary evidence sources were used to contextualize these findings, summarize contemporary clinical concepts and consensus-based recommendations, and address areas in which primary pediatric evidence remains limited. Where secondary publications incorporated primary studies that were also included in the present review, their conclusions were not treated as independent evidence, thereby minimizing the potential for double-counting of overlapping data.

The results were organized into thematic sections addressing the principal components of contemporary pediatric SBS management, including intestinal adaptation, nutritional rehabilitation, predictors of enteral autonomy, pharmacological advances, surgical reconstruction therapies, IRPs and intestinal transplantation. Particular emphasis was placed on the interaction between nutritional, medical, and surgical management strategies and their role within multidisciplinary intestinal rehabilitation programs [4,8,22].

### 2.5. Methodological Quality and Risk-of-Bias Assessment

The methodological quality and risk of bias of the primary studies included in the evidence synthesis were evaluated using design-specific critical appraisal instruments developed by the Joanna Briggs Institute (JBI). The appropriate JBI tool was selected according to the methodological design of each study, allowing cohort studies, case series, cross-sectional studies, and interventional studies to be assessed against criteria specific to their respective designs. This approach was adopted to account for the methodological heterogeneity of the primary evidence base while maintaining a structured and transparent appraisal process.

Methodological limitations identified during critical appraisal were considered qualitatively when interpreting the consistency, strength, and clinical applicability of the available evidence. No study was excluded solely on the basis of methodological quality. Given the heterogeneity of study designs and the absence of a directly comparable summary score across the design-specific appraisal instruments, the assessments were not combined into an aggregate numerical risk-of-bias score.

Secondary evidence sources, including narrative, systematic, or integrative reviews and evidence-based position papers, were not included in the primary methodological appraisal because they were used exclusively for contextual interpretation and for describing contemporary recommendations rather than as independent sources of primary outcome data.

## 3. Results

### 3.1. Study Selection and Evidence Base

The electronic database searches identified 305 records. No duplicate records were identified; therefore, all 305 records underwent title and abstract screening. Following exclusion of 250 records at this stage, 55 full-text publications were assessed for eligibility. Twenty-five full-text publications were subsequently excluded, resulting in 30 clinically relevant publications retained for the qualitative synthesis (Figure 1).

The characteristics of the 30 publications retained for the qualitative synthesis are summarized in Supplementary Table S1 according to publication type or study design, population or scope, principal management domain, and main contribution to the synthesis.

The evidence base comprised both primary clinical studies and secondary publications. Primary studies included observational cohort studies, registry-based analyses, clinical case series, interventional studies, and survey-based research, and constituted the principal evidence base for the qualitative synthesis of clinical outcomes and management strategies. Secondary publications, including narrative, systematic, or integrative reviews and an evidence-based position paper, were retained separately to contextualize the primary findings and summarize contemporary concepts and recommendations. The methodological PRISMA reference was used exclusively for reporting purposes and was not considered part of the clinical evidence base. Quantitative meta-analysis was not undertaken because of substantial heterogeneity in study design, SBS etiology, residual bowel anatomy, age at intestinal resection, therapeutic interventions, follow-up duration, and reported outcome definitions.

The methodological appraisal of the primary studies revealed variability in the strength of the available evidence, largely reflecting the heterogeneity of the underlying study designs. The principal methodological limitations were related to the predominance of retrospective and observational evidence, relatively small pediatric cohorts, variability in follow-up duration, and limited adjustment for potential confounding factors. Although the available interventional studies provided comparatively stronger evidence for specific therapeutic strategies, their interpretation remained constrained by small sample sizes and the limited availability of controlled pediatric data. These methodological considerations were taken into account throughout the qualitative synthesis, particularly when interpreting the consistency and clinical applicability of the reported associations and treatment effects.

### 3.2. Intestinal Adaptation as the Foundation of Surgical and Medical Management

All medical and surgical approaches are based on the biological foundation of intestinal adaptation, which is a key factor in determining outcomes in pediatric SBS [8]. Bowel dilatation, crypt hyperplasia, villus enlargement, altered motility, and higher absorptive capacity are among the structural and functional alterations linked to adaptation [3,8]. According to clinical evaluations, adaptation is most noticeable in the first 12 to 24 months after resection, especially in newborns and infants [8,12].

From a surgical perspective, intestinal adaptation is generally beneficial and remains the desired biological response after bowel resection. However, in some patients, progressive bowel dilatation may be associated with dysmotility, dysbiosis or small intestinal bacterial overgrowth, impaired absorption, feeding intolerance, and persistent dependence on parenteral support [23]. Therefore, surgical evaluation should focus on identifying patients in whom bowel dilatation reflects poor functional adaptation rather than successful rehabilitation. In carefully selected cases, particularly when dilatation is associated with dysmotility and failure to advance enteral nutrition, bowel tapering or lengthening procedures may be considered [22,25].

### 3.3. Nutritional Strategies and Their Impact on Surgical Decision-Making

The most significant factor influencing intestinal adaptation and functional recovery is generally found to be the early start and progression of enteral feeding [3,11,12]. Because of its trophic and immunomodulatory qualities, human milk is recommended for infants, especially after necrotizing enterocolitis [3]. Individualized feeding plans should balance bolus feeding, which promotes physiological hormonal responses and intestinal motility, with continuous feeding, which may improve tolerance during the early stages of rehabilitation [11,26].

Gastrostomy feeding has been evaluated as an adjunct to intestinal rehabilitation. Available evidence suggests that gastrostomy placement may facilitate nutritional advancement and improve feeding tolerance without increasing complication rates, supporting its role in optimizing enteral nutrition during rehabilitation. In carefully selected patients who fail to achieve adequate intestinal adaptation despite optimized nutritional management, gastrostomy may also help maintain nutritional status while ongoing multidisciplinary evaluation determines whether further medical or surgical intervention is warranted [27].

### 3.4. Parenteral Support–Related Complications and Surgical Implications

In pediatric SBS, chronic reliance on PS continues to be a significant cause of morbidity. The most clinically important complications include IFALD and catheter-related bloodstream infections (CRBSIs) [4,23]. According to registry data, the primary causes of surgical escalation and transplant referral are gradual loss of central venous access and recurrent CRBSIs [9].

Vitamin B12, vitamin D, iron, and fat-soluble vitamin deficiencies are common according to retrospective investigations and may negatively affect wound healing, immune function, and postoperative recovery [13].

Metabolic bone disease is increasingly recognized as a long-term complication of pediatric SBS. An integrative review reported reduced bone mineral density in a substantial proportion of patients, supporting routine dual-energy X-ray absorptiometry (DEXA) surveillance as part of comprehensive SBS care, particularly in children undergoing major reconstructive surgery [14].

### 3.5. Predictors of Enteral Autonomy and Risk Stratification

Residual small-bowel length was consistently identified as the principal anatomical predictor of enteral autonomy, while preservation of the colon in continuity was also associated with more favorable rehabilitation outcomes [3,15,28]. Cohort data further indicate that enteral autonomy can be achieved in a substantial proportion of children with neonatal-onset SBS, although the probability and timing of autonomy vary according to residual anatomy and clinical course [8,15].

Predictive approaches incorporating both anatomical and clinical characteristics have been developed to estimate the likelihood and timing of enteral autonomy. Longer residual bowel length, preserved colonic continuity, and measures of functional adaptation have been associated with more favorable outcomes, whereas disease-severity models may provide additional support for risk stratification [24,28].

### 3.6. Pharmacologic Advances and Their Potential Implications for Surgical Decision-Making

A major pharmacological development in the management of pediatric SBS has been the introduction of glucagon-like peptide-2 (GLP-2) analog therapy. Teduglutide has been evaluated in infants and children with SBS-associated intestinal failure who remain dependent on parenteral support, with pediatric clinical studies and real-world evidence demonstrating clinically meaningful reductions in parenteral support requirements in a proportion of treated patients [16,19].

These findings support a role for teduglutide as part of individualized intestinal rehabilitation in appropriately selected patients. However, the currently available pediatric evidence primarily addresses reductions in parenteral support requirements and achievement of enteral autonomy and does not establish whether teduglutide reduces the subsequent need for autologous bowel reconstruction or intestinal transplantation. Any potential influence of GLP-2 analog therapy on the timing or likelihood of surgical intervention should therefore be regarded as hypothetical and requires prospective evaluation [7,16,19].

Additionally, recent evidence has expanded clinical experience with teduglutide in younger patients. In an analysis of two phase III studies and one extension study, Chiba et al. evaluated teduglutide in infants and children with SBS-associated intestinal failure. After 24 weeks of treatment, a ≥20% reduction in parenteral support requirements from baseline was achieved in 57.1% of infants and 66.7% of children receiving teduglutide. Among patients continuing treatment, 50.0% of infants and 80.0% of children maintained a ≥20% reduction in parenteral support requirements at 48 weeks. Two children achieved enteral autonomy after 12 and 28 weeks of treatment, respectively. Teduglutide was generally well tolerated, with adverse events consistent with the underlying disease and the known safety profile of the treatment [19].

### 3.7. Surgical Rehabilitation Techniques

#### 3.7.1. Restoration of Intestinal Continuity

Restoration of intestinal continuity should be considered early in the rehabilitation pathway whenever technically feasible, particularly when a substantial segment of distal small bowel and/or colon remains excluded from the enteric stream. Re-establishing continuity increases the functional absorptive surface, restores the contribution of the colon to fluid and electrolyte absorption and energy salvage, and may facilitate intestinal adaptation and reduction in parenteral support requirements [3,13,29]. Patient selection should be based on residual small-bowel length and anatomy, the availability and functional integrity of the distal bowel and colon, the presence of the ileocecal valve, anastomotic feasibility, and the absence of contraindications such as active intra-abdominal sepsis or severe clinical instability. Timing should therefore be individualized according to postoperative recovery and anatomical feasibility rather than determined by a fixed interval after the initial resection. When feasible, restoration of continuity should generally precede consideration of bowel-lengthening procedures because functional recruitment of excluded bowel may itself improve enteral tolerance and reduce parenteral support requirements [3,13,23,29].

#### 3.7.2. Autologous Bowel Reconstruction

Autologous bowel reconstruction should not be indicated on the basis of bowel dilatation alone. Persistent or progressive dilatation should instead prompt a comprehensive assessment of intestinal function within a multidisciplinary intestinal rehabilitation program. Appropriate candidates are patients in whom dilated bowel is associated with clinically relevant functional impairment, including objective or persistent evidence of dysmotility, recurrent small intestinal bacterial overgrowth (SIBO), feeding intolerance, failure to advance enteral nutrition, poor growth, or persistent parenteral support dependence despite optimized medical and nutritional rehabilitation [3,13,23,28,29]. Surgical assessment should additionally incorporate residual small-bowel length, bowel diameter and distribution of dilatation, continuity of the colon, presence of the ileocecal valve, mesenteric vascular anatomy, previous abdominal procedures, and the trajectory of nutritional adaptation. This distinction is clinically important because bowel dilatation may represent part of the adaptive response to intestinal resection and does not, in isolation, constitute an indication for reconstructive surgery.

Among patients meeting these functional and anatomical criteria, selection between STEP and LILT should be individualized. STEP is generally more broadly applicable in patients with sufficiently dilated bowel and is technically less demanding, does not require division of the mesentery, and can be repeated when recurrent dilatation occurs. LILT is more anatomically restrictive and is best suited to highly selected patients with uniformly and markedly dilated bowel of sufficient length and favorable mesenteric vascular anatomy to permit longitudinal division while preserving adequate perfusion of both hemiloops [3,13,29]. Consequently, the choice between STEP and LILT should reflect bowel morphology and mesenteric anatomy, previous surgical history, the functional objectives of reconstruction, and institutional expertise rather than a predetermined preference for either procedure.

Timing of autologous bowel reconstruction should similarly be individualized. Lengthening procedures should generally be considered only after an adequate period of multidisciplinary intestinal rehabilitation has allowed assessment of the patient’s adaptive potential and nutritional trajectory. Earlier intervention may be appropriate when progressive dilatation is accompanied by clinically significant dysmotility, recurrent SIBO, inability to advance enteral feeding, or worsening complications of prolonged parenteral support despite optimized therapy. Conversely, stable patients demonstrating continued enteral progression and reduction in parenteral support requirements may benefit from continued rehabilitation, even in the presence of bowel dilatation [3,13,23,29].

The principal surgical strategies used in pediatric SBS, together with the main considerations guiding patient selection, potential advantages, and procedure-specific limitations, are summarized in Table 1.

The table summarizes the main surgical procedures currently used in pediatric SBS, including indications, advantages, and major limitations.

#### 3.7.3. Timing and Patient Selection

Surgical timing in pediatric SBS should be determined by the interaction between residual anatomy, functional bowel performance, nutritional progression, and complications arising during intestinal rehabilitation rather than by bowel length or dilatation alone. Serial multidisciplinary assessment should therefore consider the trajectory of enteral tolerance and parenteral support requirements, growth, bowel morphology, clinically significant dysmotility, recurrent SIBO, and the development of parenteral support–related morbidity. Restoration of intestinal continuity should be pursued when anatomically feasible once the patient is clinically suitable for reconstruction, whereas STEP or LILT should be reserved for patients with functionally significant dilated bowel who fail to demonstrate satisfactory progression despite optimized rehabilitation. Importantly, persistent dilatation in a clinically stable child who continues to advance enteral nutrition and reduce parenteral support does not, by itself, justify bowel-lengthening surgery [3,13,23,29].

Accordingly, surgical intervention should not be triggered by a single anatomical threshold or an isolated clinical finding. The decision should instead reflect a longitudinal assessment of whether the remaining bowel is undergoing effective adaptation or whether maladaptive dilatation and impaired intestinal function are preventing further nutritional progression. This approach seeks to avoid both premature reconstruction, which may interrupt ongoing adaptation, and excessive delay in patients with persistent functional impairment or accumulating complications of prolonged parenteral support [3,9,23,29].

#### 3.7.4. Intestinal Transplantation

Intestinal transplantation represents a rescue strategy rather than a routine step in the surgical management of pediatric SBS. Improvements in multidisciplinary intestinal rehabilitation have substantially narrowed its indications, and residual bowel length or persistent dependence on parenteral support alone should not be regarded as sufficient indications for transplantation. Referral should be considered in carefully selected patients who develop progressive and potentially irreversible intestinal failure–associated liver disease (IFALD), recurrent life-threatening catheter-related bloodstream infections despite optimized line care, or progressive loss of central venous access that threatens the feasibility of long-term parenteral support despite optimized intestinal rehabilitation [3,12,23].

The timing of referral is particularly important and should precede the development of advanced irreversible complications that may compromise transplant candidacy. Transplant assessment should therefore occur within an experienced multidisciplinary intestinal rehabilitation and transplant program when the probability of safely maintaining long-term parenteral support is declining despite optimized medical, nutritional, pharmacological, and reconstructive strategies. Conversely, transplantation should not be pursued solely on the basis of very short residual bowel length, because long-term survival and, in selected patients, meaningful intestinal rehabilitation remain possible even in ultrashort bowel syndrome [3,8,15,23].

### 3.8. Emerging and Underexplored Domains

Recent evidence suggests that the intestinal microbiome may play a significant role in intestinal adaptation following bowel resection and has emerged as a promising target for future therapeutic strategies in pediatric SBS. Piper et al. demonstrated that infants with small bowel stomas exhibit distinct microbial and metabolomic profiles characterized by reduced bacterial diversity and alterations in metabolic pathways compared with healthy controls, suggesting that postoperative dysbiosis may contribute to intestinal inflammation and impaired adaptation [18]. These findings support the hypothesis that the intestinal microbiome actively participates in the adaptive process rather than merely reflecting disease severity.

Although modulation of the intestinal microbiota through probiotic supplementation has been proposed as a potential therapeutic strategy, current evidence remains insufficient to support its routine clinical use. Piper et al. reported that *Lactobacillus* supplementation altered the intestinal microbial composition; however, these microbiological changes were not accompanied by consistent improvements in clinical or nutritional outcomes [30]. Similarly, current reviews conclude that the available evidence is limited by small sample sizes, heterogeneous probiotic formulations, and the lack of randomized controlled trials, precluding evidence-based recommendations for routine probiotic administration in children with short bowel syndrome [31].

## 4. Discussion

This systematic review synthesizes contemporary primary clinical evidence on the management of children with SBS following intestinal resection and contextualizes these findings within the broader framework of current secondary evidence and expert recommendations. Collectively, the available evidence suggests that improvements in pediatric SBS outcomes have resulted primarily from the coordinated integration and appropriate sequencing of nutritional, pharmacological, and surgical strategies within multidisciplinary intestinal rehabilitation programs, rather than from any single therapeutic intervention [4,9,10].

### 4.1. Intestinal Rehabilitation Programs as the Central Organizing Principle

The development of dedicated IRPs has fundamentally changed the management of pediatric SBS. Current evidence demonstrates that multidisciplinary care involving pediatric surgeons, gastroenterologists, nutrition specialists, pharmacists, specialized nurses, and other allied healthcare professionals is associated with improved survival, higher rates of enteral autonomy, fewer catheter-related complications, and a reduced need for intestinal transplantation [3,9,12,23]. Rather than functioning solely as nutritional support programs, IRPs provide a structured framework for the continuous assessment of intestinal adaptation and allow treatment strategies to evolve according to each patient’s clinical course.

From a surgical perspective, IRPs facilitate careful longitudinal evaluation of bowel function, nutritional progress, and intestinal morphology, allowing early recognition of patients who are unlikely to achieve adequate adaptation with conservative management alone. This multidisciplinary approach supports appropriate patient selection and optimal timing for bowel reconstruction, while avoiding both unnecessary early surgery and delayed intervention in patients who develop progressive bowel dilatation, dysmotility, or recurrent septic complications [3,13,29].

**Figure 2:**
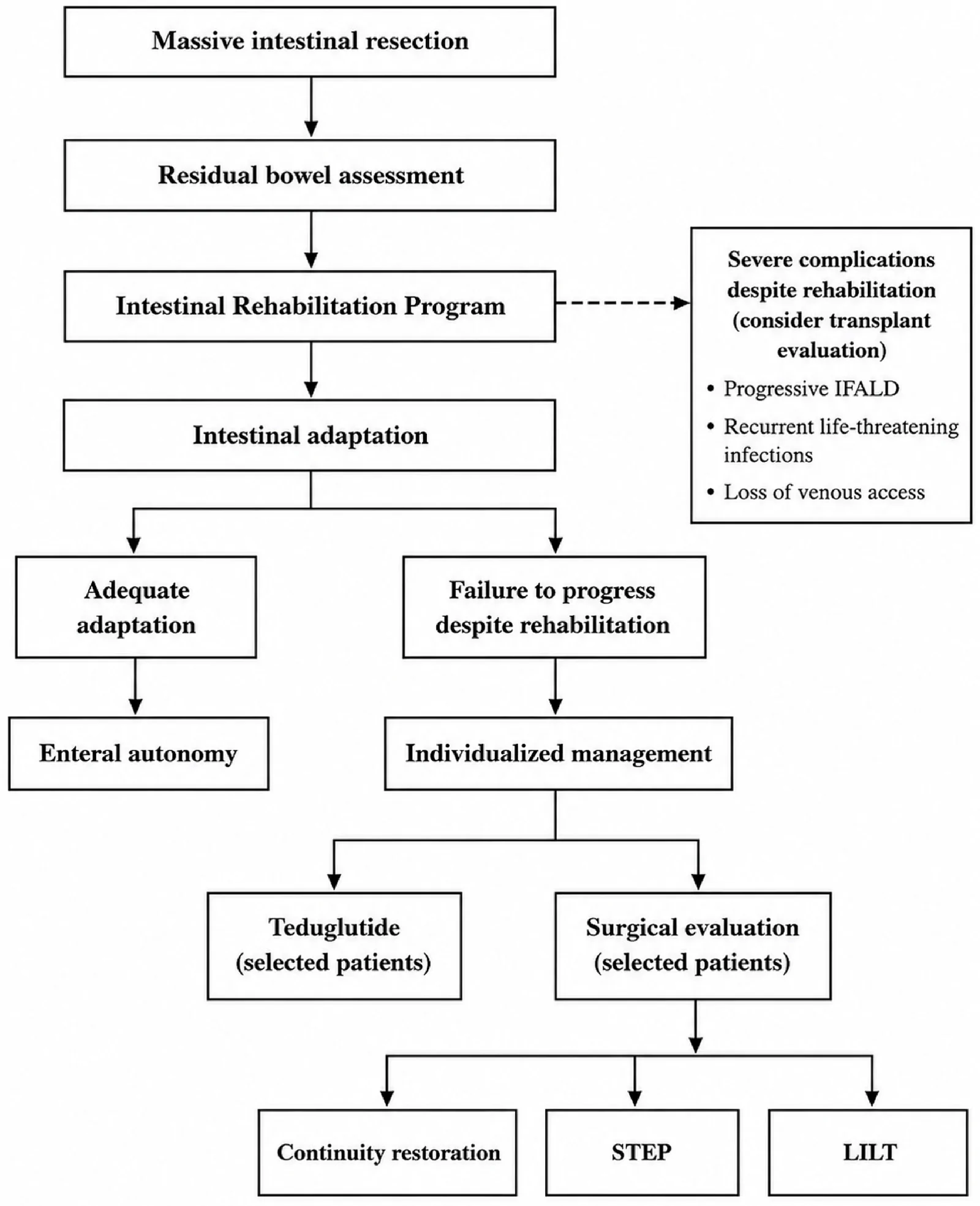
Proposed multidisciplinary management pathway for pediatric short bowel syndrome. The pathway illustrates progression from intestinal rehabilitation and adaptation toward enteral autonomy or individualized medical and surgical management. A separate pathway indicates consideration of intestinal transplant evaluation in patients who develop severe complications despite optimized intestinal rehabilitation, including progressive IFALD, recurrent life-threatening infections, or loss of central venous access.

The multidisciplinary approach proposed throughout this review is illustrated in Figure 2, highlighting the integration of nutritional optimization, pharmacological therapy, surgical intervention, and long-term rehabilitation within contemporary intestinal rehabilitation programs.

### 4.2. Predictors of Adaptation and Surgical Decision-Making

The findings of this review reinforce the concept that surgical decision-making in pediatric SBS cannot be based on a single anatomical variable. Although residual small-bowel length and colon continuity remain important predictors of enteral autonomy, their clinical significance depends on the functional performance of the remnant intestine and the trajectory of adaptation. Consequently, anatomical characteristics should be interpreted together with enteral tolerance, growth, parenteral support requirements, bowel morphology and motility, and complications arising during rehabilitation [3,15,23,28,29].

This distinction is particularly relevant when evaluating bowel dilatation. Dilatation may accompany physiological adaptation after intestinal resection, whereas progressive dilatation associated with dysmotility, recurrent SIBO, feeding intolerance, or failure of nutritional progression may indicate maladaptive intestinal function. Accordingly, bowel diameter alone should not determine the indication or timing of reconstructive surgery. Rather, serial multidisciplinary assessment is required to distinguish patients who continue to benefit from conservative rehabilitation from those in whom functional impairment is preventing further progress [3,13,23,29].

The same principle applies to children with very limited residual bowel length. Favorable outcomes reported in selected patients with neonatal-onset and ultrashort bowel syndrome indicate that severe anatomical loss does not invariably predict failure of rehabilitation. These observations support individualized longitudinal assessment rather than fixed bowel-length thresholds for surgical escalation or transplant referral [3,8,15].

The principal anatomical and clinical factors associated with enteral autonomy and their implications for individualized multidisciplinary management are summarized in Table 2.

The factors summarized above contribute to individualized multidisciplinary management but should not be interpreted as isolated indications for surgical intervention.

### 4.3. Nutritional Optimization and Its Surgical Implications

Nutritional progression represents both a therapeutic objective and an important functional indicator of intestinal adaptation. Although optimization of enteral nutrition remains fundamental to intestinal rehabilitation, its relevance to surgical decision-making lies primarily in the longitudinal response to therapy rather than in any individual feeding strategy. Persistent feeding intolerance, poor growth, inability to advance enteral nutrition, or continued high parenteral support requirements despite optimized rehabilitation may indicate inadequate functional adaptation and should prompt multidisciplinary reassessment [2,3,22,23].

Importantly, failure of nutritional progression should not automatically trigger surgical intervention. Rather, it should prompt evaluation for potentially correctable anatomical or functional factors, including bowel dilatation, dysmotility, mechanical obstruction, or recurrent SIBO. Nutritional trajectory should therefore be interpreted together with residual bowel anatomy and functional performance when determining whether continued rehabilitation or surgical evaluation is appropriate [3,22,23,29].

### 4.4. Parenteral Support–Related Morbidity as a Driver of Surgical Escalation

Although advances in PN have substantially improved the survival of children with SBS, long-term dependence on PS remains associated with significant complications that frequently influence surgical decision-making. IFALD, CRBSIs, and progressive loss of central venous access continue to represent major causes of morbidity and are among the principal indications for considering surgical escalation or referral for intestinal transplantation [4,9,23].

From a surgical perspective, these findings support a proactive rather than reactive approach to intestinal rehabilitation. Surgical evaluation should not be postponed until irreversible complications of prolonged PN have developed. Instead, reconstructive procedures should be considered within the broader framework of multidisciplinary intestinal rehabilitation in patients with bowel dilatation and associated complications such as SIBO, balancing the potential for further intestinal adaptation against the cumulative risks of prolonged PS [3,9,23].

### 4.5. Pharmacological Intestinotrophic Therapy and Its Impact on Surgical Strategy

From a surgical perspective, the demonstrated reduction in parenteral support requirements with teduglutide may influence the longitudinal assessment of intestinal adaptation and nutritional progression in selected patients. However, evidence that GLP-2 analog therapy alters the indication for delays or reduces the need for STEP, LILT, or intestinal transplantation is currently lacking. Accordingly, teduglutide should not be considered a substitute for indicated reconstructive surgery, nor should surgical intervention be deferred solely on the basis of pharmacological therapy. Decisions regarding reconstructive surgery or transplantation should continue to be based on residual anatomy, bowel function, nutritional progression, complications of intestinal failure and parenteral support, and response to multidisciplinary rehabilitation. Further prospective studies are required to determine whether teduglutide has any measurable effect on subsequent surgical requirements [7,16,19].

### 4.6. Role of Surgical Rehabilitation in the Contemporary Era

Autologous bowel reconstruction remains an important component of intestinal rehabilitation in carefully selected children with SBS who fail to achieve satisfactory functional and nutritional progression despite optimized medical and nutritional therapy. Importantly, bowel dilatation should not be interpreted as an isolated indication for surgery. Rather, reconstructive intervention should be considered when persistent or progressive dilatation is accompanied by clinically significant dysmotility, recurrent SIBO, feeding intolerance, failure to advance enteral nutrition, poor growth, or persistent parenteral support dependence, after residual bowel anatomy and the trajectory of intestinal adaptation have been comprehensively reassessed [3,13,23,29].

The two most established autologous bowel-lengthening procedures are LILT and STEP. Both techniques have demonstrated favorable outcomes in appropriately selected patients, including improved enteral tolerance, progressive reduction in PN requirements, and increased rates of enteral autonomy. However, available evidence does not demonstrate the superiority of either procedure, and surgical decision-making should therefore be individualized according to residual bowel anatomy, degree of bowel dilatation, mesenteric vascular anatomy, previous abdominal surgery, and the expertise of specialized intestinal rehabilitation centers [3,13,29].

Both procedures are associated with procedure-specific limitations and potential complications that should be considered during patient selection. LILT is technically demanding and highly dependent on favorable bowel and mesenteric anatomy, limiting its applicability to carefully selected patients. In contrast, STEP is technically less complex and can be repeated when necessary; however, alteration of bowel geometry may affect intestinal motility, and complications such as staple-line ulcers, bleeding, bowel obstruction, or the need for repeat lengthening procedures have been reported. Consequently, procedure selection should account for both anatomical suitability and the specific risk profile of each technique and should be undertaken within experienced multidisciplinary intestinal rehabilitation programs [13,29].

### 4.7. Emerging Domains and Future Directions

Recent advances in microbiome research have highlighted the potential role of intestinal microbial composition in modulating intestinal adaptation following bowel resection. Infants with small bowel stomas demonstrate distinct microbial and metabolomic profiles, suggesting that postoperative dysbiosis may contribute to intestinal inflammation, impaired nutrient absorption, and variability in adaptive capacity. Nevertheless, despite growing interest in microbiome-targeted therapies, current evidence remains insufficient to support the routine use of probiotics or other microbiota-modulating interventions in clinical practice, and their role in pediatric intestinal rehabilitation requires further investigation [18,30,31].

Future research should prioritize well-designed prospective multicenter pediatric studies with standardized outcome measures and long-term follow-up to better define the optimal integration of nutritional, pharmacological, and surgical therapies. Particular attention should be directed toward identifying reliable predictors of enteral autonomy, optimizing patient selection for bowel reconstruction and GLP-2 analog therapy, and evaluating the long-term impact of emerging therapeutic strategies, including microbiome modulation, within multidisciplinary IRPs.

### 4.8. Limitations

The findings of this review should be interpreted in light of several limitations. The primary evidence base is methodologically heterogeneous and consists predominantly of observational evidence, with substantial variation in study design, patient populations, underlying etiologies, residual bowel anatomy, therapeutic strategies, follow-up duration, and reported outcome definitions. These differences limit direct between-study comparisons and preclude meaningful quantitative pooling. Furthermore, prospective controlled studies remain scarce in pediatric SBS, restricting the strength of causal inference regarding several therapeutic strategies. Secondary reviews and position papers were therefore used to provide clinical context and summarize contemporary recommendations but were not considered independent sources of primary clinical evidence.

An additional limitation is the absence of universally standardized definitions for several clinically important outcomes, including intestinal failure, enteral autonomy, and treatment success. Variability in outcome definitions and reporting further complicates comparisons across studies and limits the certainty with which the relative effectiveness of different rehabilitation strategies can be established. Publication bias also cannot be excluded, particularly in an evidence base dominated by observational studies and relatively small pediatric cohorts.

Despite these limitations, the available evidence consistently supports the central role of multidisciplinary intestinal rehabilitation in the management of pediatric SBS. Nevertheless, well-designed prospective multicenter studies using standardized definitions, clinically meaningful outcome measures, and sufficiently long follow-up are required to better define the relative contributions and optimal sequencing of nutritional, pharmacological, and surgical interventions.

## 5. Conclusions

Pediatric SBS remains one of the most challenging conditions in pediatric surgery, requiring long-term multidisciplinary management and individualized treatment strategies. Although significant progress has been made over the past two decades, successful rehabilitation still depends on achieving the best possible balance between nutritional support, intestinal adaptation, pharmacological therapy, and appropriately timed surgical intervention.

The evidence reviewed highlights the importance of dedicated IRPs, which have transformed the care of these patients by improving survival, increasing the likelihood of enteral autonomy, and reducing the need for intestinal transplantation. At the same time, advances in pharmacological therapy, particularly GLP-2 analogs, and refinements in autologous bowel reconstruction have broadened the therapeutic options available for children with complex intestinal failure; however, the extent to which pharmacological therapy modifies subsequent surgical requirements remains uncertain.

Despite these advances, many aspects of pediatric SBS management remain incompletely understood. Future research should focus on prospective multicenter studies, standardized outcome measures, and long-term evaluation of both medical and surgical therapies. A better understanding of intestinal adaptation and improved tools for predicting enteral autonomy will help clinicians make more informed decisions and continue to improve the long-term outcomes and quality of life of children living with short bowel syndrome.

## Figures and Tables

**Table 1:** Current surgical strategies for pediatric short bowel syndrome: patient-selection considerations, potential advantages, and limitations.

Procedure	Main Patient-Selection Considerations	Advantages	Limitations
**Restoration of intestinal continuity**	Available and functionally suitable distal bowel and/or colon that can be safely restored to continuity	Improves fluid and electrolyte absorption, promotes intestinal adaptation, and may reduce dependence on parenteral support	Feasible only when residual bowel anatomy allows restoration of continuity
**STEP**	Functionally significant bowel dilatation associated with dysmotility, recurrent SIBO, feeding intolerance, or failure of nutritional progression despite optimized intestinal rehabilitation	Technically less demanding than LILT, repeatable when appropriate, increases functional bowel length	May alter bowel motility due to disruption of muscle fiber orientation; staple-line/perianastomotic ulcers have been reported; long-term outcomes depend on patient selection and some patients require repeat procedures
**LILT (Bianchi)**	Marked, relatively uniform bowel dilatation with favorable bowel length and mesenteric vascular anatomy in a patient with persistent functional impairment despite optimized rehabilitation	Preserves bowel physiology and increases absorptive surface	Technically demanding, anatomy dependent, performed only in highly selected patients
**Intestinal transplantation**	Progressive potentially irreversible IFALD, recurrent life-threatening CRBSIs, or progressive loss of central venous access threatening the feasibility of long-term parenteral support despite optimized intestinal rehabilitation	Life-saving treatment for carefully selected patients in whom long-term parenteral nutrition is no longer feasible	Lifelong immunosuppression, risk of graft rejection, infectious complications, post-transplant lymphoproliferative disease, significant morbidity, limited donor availability, and outcomes that may vary according to graft type, recipient characteristics, center experience, and transplant era.

**Table 2:** Major predictors of enteral autonomy and their implications for multidisciplinary management in pediatric short bowel syndrome.

Predictor	Clinical Significance	Clinical Relevance	Key References
**Residual small-bowel length**	Strongest anatomical predictor of intestinal adaptation	Supports prognosis and helps identify patients likely to benefit from prolonged intestinal rehabilitation before considering further interventions	[3,15,28]
**Colon in continuity**	Improves fluid and electrolyte absorption and energy salvage	Restoration of intestinal continuity should be considered whenever anatomically feasible as part of intestinal rehabilitation	[3,23,29]
**Ileocecal valve**	Reduces rapid intestinal transit and the risk of small intestinal bacterial overgrowth	Preservation of the ileocecal valve favors intestinal adaptation and conservative rehabilitation	[3,23]
**Enteral nutrition tolerance**	Functional marker of intestinal adaptation	Persistent failure to advance enteral feeding may indicate the need for further evaluation, including assessment for bowel dilatation and dysmotility	[2,22,23]
**Response to teduglutide**	Associated with reduced parenteral support requirements in responsive patients	May support continued intestinal rehabilitation and reassessment of parenteral support needs; its effect on subsequent surgical requirements remains uncertain	[7,16,19]
**Disease severity score**	Estimates the likelihood of achieving enteral autonomy	Supports individualized risk stratification and multidisciplinary treatment planning	[24,28]

## Data Availability

No new data were created or analyzed in this study. Data sharing is not applicable to this article.

## Supplementary Materials

The following supporting information can be downloaded at: https://www.mdpi.com/article/10.3390/jcm15187291/s1, Table S1: Characteristics of the 30 publications included in the qualitative synthesis.

## Abbreviations

The following abbreviations are used in this manuscript:

SBS	Short Bowel Syndrome
PS	Parenteral Support
IRP	Intestinal Rehabilitation Program
IFALD	Intestinal Failure–Associated Liver Disease
CRBSI	Catheter-Related Bloodstream Infections
GLP-2	Glucagon-Like Peptide-2
STEP	Serial Transverse Enteroplasty
LILT	Longitudinal Intestinal Lengthening and Tailoring

## References

[1] Ou J., Courtney C.M., Steinberger A.E., Tecos M.E., Warner B.W. (2020). Nutrition in Necrotizing Enterocolitis and Following Intestinal Resection. Nutrients.

[2] Channabasappa N., Girouard S., Nguyen V., Piper H. (2020). Enteral Nutrition in Pediatric Short-Bowel Syndrome. Nutr. Clin. Pract..

[3] Belza C., Wales P.W. (2022). Management of pediatric intestinal failure related to short bowel syndrome. Semin. Pediatr. Surg..

[4] Phelps H.M., Warner B.W. (2023). Intestinal adaptation and rehabilitation. Semin. Pediatr. Surg..

[5] Sukhotnik I., Levi R., Moran-Lev H. (2023). Impact of Dietary Protein on the Management of Pediatric Short Bowel Syndrome. Nutrients.

[6] Caporilli C., Giannì G., Grassi F., Esposito S. (2023). An Overview of Short-Bowel Syndrome in Pediatric Patients: Focus on Clinical Management and Prevention of Complications. Nutrients.

[7] El Khatib M., Billiauws L., Joly F. (2023). The indications and results of the use of teduglutide in patients with short bowel. Curr. Opin. Clin. Nutr. Metab. Care.

[8] Batra A., Keys S.C., Johnson M.J., Wheeler R.A., Beattie R.M. (2017). Epidemiology, management and outcome of ultrashort bowel syndrome in infancy. Arch. Dis. Childhood. Fetal Neonatal Ed..

[9] Tannuri U., Barros F., Tannuri A.C. (2016). Treatment of short bowel syndrome in children. Value of the Intestinal Rehabilitation Program. Rev. Assoc. Médica Bras..

[10] Cohran V.C., Prozialeck J.D., Cole C.R. (2017). Redefining short bowel syndrome in the 21st century. Pediatr. Res..

[11] Eshel Fuhrer A., Moran-Lev H., Dranitzki Y., Ben-Shahar Y., Sukhotnik I. (2022). The role of gastrostomy feeding during intestinal rehabilitation for children with short bowel syndrome. Pediatr. Surg. Int..

[12] Totonelli G., Tambucci R., Boscarelli A., Hermans D., Dall’Oglio L., Diamanti A., d’Aische A.D.B., Pakarinen M., Reding R., Morini F. (2018). Pediatric Intestinal Rehabilitation and Transplantation Registry: Initial Report from a European Collaborative Registry. Eur. J. Pediatr. Surg..

[13] Dore M., Junco P.T., Andres A.M., Sánchez-Galán A., Amesty M.V., Ramos E., Prieto G., Hernandez F., Lopez Santamaria M. (2016). Surgical Rehabilitation Techniques in Children with Poor Prognosis Short Bowel Syndrome. Eur. J. Pediatr. Surg..

[14] Blum A.G.R., Russo T.D.H., Nogueira R.J.N. (2023). Dual X-ray absorptiometry monitoring in pediatric short bowel syndrome: An integrative review. Rev. Paul. Pediatr..

[15] Fatemizadeh R., Gollins L., Hagan J., Debuyserie A., King K., Vogel A.M., Van Buren K.L., Hair A.B., Premkumar M.H. (2022). In neonatal-onset surgical short bowel syndrome survival is high, and enteral autonomy is related to residual bowel length. J. Parenter. Enter. Nutr..

[16] Rosete B.E., Wendel D., Horslen S.P. (2021). Teduglutide for pediatric short bowel syndrome patients. Expert Rev. Gastroenterol. Hepatol..

[17] Chatzidaki V., Wood R., Alegakis A., Lawson M., Fagbemi A. (2023). Parenteral support and micronutrient deficiencies in children with short bowel syndrome: A comprehensive retrospective study. Clin. Nutr. ESPEN.

[18] Piper H.G., Bording-Jorgensen M., Veniamin S., Zhang Z., Suarez R.G., Armstrong H., Silverman J.A., Wine E. (2024). Intestinal microbial and metabolite profile in infants with small bowel stomas after bowel resection. J. Pediatr. Gastroenterol. Nutr..

[19] Chiba M., Masumoto K., Kaji T., Matsuura T., Morii M., Fagbemi A., Hill S., Pakarinen M.P., Protheroe S., Urs A. (2023). Efficacy and Safety of Teduglutide in Infants and Children with Short Bowel Syndrome Dependent on Parenteral Support. J. Pediatr. Gastroenterol. Nutr..

[20] Dariel A., Faure A., Martinez L., Morini F., Pini Prato A., Friedmacher F., Coste M.E. (2021). European Pediatric Surgeon’ Association Survey on the Management of Short-Bowel Syndrome. Eur. J. Pediatr. Surg..

[21] Liberati A., Altman D.G., Tetzlaff J., Mulrow C., Gøtzsche P.C., Ioannidis J.P., Clarke M., Devereaux P.J., Kleijnen J., Moher D. (2009). The PRISMA statement for reporting systematic reviews and meta-analyses of studies that evaluate health care interventions: Explanation and elaboration. J. Clin. Epidemiol..

[22] Premkumar M.H., Soraisham A., Bagga N., Massieu L.A., Maheshwari A. (2022). Nutritional Management of Short Bowel Syndrome. Clin. Perinatol..

[23] Norsa L., Goulet O., Alberti D., DeKooning B., Domellöf M., Haiden N., Hill S., Indrio F., Köglmeier J., Lapillonne A. (2023). Nutrition and Intestinal Rehabilitation of Children with Short Bowel Syndrome: A Position Paper of the ESPGHAN Committee on Nutrition. Part 1: From Intestinal Resection to Home Discharge. J. Pediatr. Gastroenterol. Nutr..

[24] Gonzalez-Hernandez J., Prajapati P., Ogola G., Channabasappa N., Drews B., Piper H.G. (2017). Predicting time to full enteral nutrition in children after significant bowel resection. J. Pediatr. Surg..

[25] Capriati T., Giorgio D., Fusaro F., Candusso M., Schingo P., Caldaro T., Laureti F., Elia D., Diamanti A. (2018). Pediatric Short Bowel Syndrome: Predicting Four-Year Outcome after Massive Neonatal Resection. Eur. J. Pediatr. Surg..

[26] Puoti M.G., Köglmeier J. (2022). Nutritional Management of Intestinal Failure due to Short Bowel Syndrome in Children. Nutrients.

[27] Chandra R., Kesavan A. (2018). Current treatment paradigms in pediatric short bowel syndrome. Clin. J. Gastroenterol..

[28] Belza C., Fitzgerald K., de Silva N., Avitzur Y., Wales P.W. (2019). Early Predictors of Enteral Autonomy in Pediatric Intestinal Failure Resulting From Short Bowel Syndrome: Development of a Disease Severity Scoring Tool. J. Parenter. Enteral. Nutr..

[29] Muto M., Kaji T., Onishi S., Yano K., Yamada W., Ieiri S. (2022). An overview of the current management of short-bowel syndrome in pediatric patients. Surg. Today.

[30] Piper H.G., Coughlin L.A., Hussain S., Nguyen V., Channabasappa N., Koh A.Y. (2020). The Impact of Lactobacillus Probiotics on the Gut Microbiota in Children with Short Bowel Syndrome. J. Surg. Res..

[31] Höllwarth M.E., Solari V. (2021). Nutritional and pharmacological strategy in children with short bowel syndrome. Pediatr. Surg. Int..

