# Supplementary material for: Updates in the Management of Short Bowel Syndrome Secondary to Intestinal Resection in the Pediatric Population
Source: J Clin Med. 2026 Sep 19;15(18):7291. doi: 10.3390/jcm15187291 (PMC13607937; doi:10.3390/jcm15187291)
Supplement: Supplementary file 1 [file jcm-15-07291-s001.zip › jcm-4456994-supplementary.pdf]

Supplementary Table S1. Characteristics of the 30 publications included in the qualitative synthesis

Ref.	Publication type / study design	Population / scope	Management domain	Main contribution to the synthesis
[1] Ou et al., 2020	Review	Neonates and children with NEC and intestinal resection	Nutritional management	Nutritional strategies following NEC and intestinal resection
[2] Channabasappa et al., 2020	Review	Children with SBS	Enteral nutrition	Enteral feeding strategies during intestinal rehabilitation
[3] Belza & Wales, 2022	Review	Pediatric SBS-associated intestinal failure	Multidisciplinary management	Contemporary principles of intestinal rehabilitation and management
[4] Phelps & Warner, 2023	Review	Pediatric patients following intestinal resection	Intestinal adaptation	Mechanisms and clinical implications of intestinal adaptation and rehabilitation
[5] Sukhotnik et al., 2023	Review	Children with SBS	Nutritional management	Role of dietary protein in nutritional rehabilitation and intestinal adaptation
[6] Caporilli et al., 2023	Review	Pediatric SBS	Multidisciplinary management	Clinical management and prevention of SBS-associated complications
[7] El Khatib et al., 2023	Review	Patients with SBS	Pharmacological therapy	Clinical indications and outcomes associated with teduglutide
[8] Batra et al., 2017	Clinical study	Infants with ultrashort bowel syndrome	Intestinal rehabilitation	Epidemiology, management, and outcomes of ultrashort bowel syndrome
[9] Tannuri et al., 2016	Clinical study	Children with SBS	Intestinal rehabilitation program	Outcomes associated with multidisciplinary intestinal rehabilitation
[10] Cohran et al., 2017	Review	Pediatric SBS	Multidisciplinary management	Evolving concepts and contemporary management of SBS
[11] Eshel Fuhrer et al., 2023	Clinical study	Children with SBS undergoing intestinal rehabilitation	Enteral nutrition	Role of gastrostomy feeding during intestinal rehabilitation

Ref.	Publication type / study design	Population / scope	Management domain	Main contribution to the synthesis
[12] Totonelli et al., 2018	Registry report	Children undergoing intestinal rehabilitation and/or transplantation	Intestinal rehabilitation and transplantation	Characteristics and outcomes within a European collaborative registry
[13] Dore et al., 2016	Clinical study	Children with poor-prognosis SBS	Surgical rehabilitation	Outcomes of autologous intestinal reconstruction in selected patients
[14] Blum et al., 2024	Integrative review	Children with SBS	Bone health and nutritional monitoring	Evidence regarding DXA assessment and bone-health monitoring
[15] Fatemizadeh et al., 2022	Clinical study	Children with neonatal-onset surgical SBS	Predictors of enteral autonomy	Relationship between residual bowel length, survival, and enteral autonomy
[16] Rosete et al., 2021	Review	Pediatric patients with SBS	Pharmacological therapy	Pediatric evidence and clinical considerations regarding teduglutide
[17] Chatzidaki et al., 2023	Retrospective study	Children with SBS	Parenteral support and nutritional monitoring	Micronutrient deficiencies during and after parenteral support
[18] Piper et al., 2024	Clinical study	Infants with small-bowel stomas following bowel resection	Intestinal microbiome	Microbial and metabolic alterations following intestinal resection
[19] Chiba et al., 2023	Interventional study	Infants and children with SBS dependent on parenteral support	Pharmacological therapy	Efficacy and safety of teduglutide and reduction in parenteral-support requirements
[20] Dariel et al., 2021	Survey	Pediatric surgeons	Surgical management	Variability in contemporary approaches to pediatric SBS management
[22] Premkumar et al., 2022	Review	Neonates and children with SBS	Nutritional management	Nutritional strategies supporting intestinal adaptation and rehabilitation
[23] Norsa et al., 2023	Evidence-based position paper	Children with SBS	Nutrition and intestinal rehabilitation	Contemporary recommendations for nutritional

Ref.	Publication type / study design	Population / scope	Management domain	Main contribution to the synthesis
				management and intestinal rehabilitation
[24] Gonzalez-Hernandez et al., 2017	Clinical study	Children following significant bowel resection	Predictors of enteral autonomy	Factors associated with time to full enteral nutrition
[25] Capriati et al., 2018	Clinical study	Children following massive neonatal intestinal resection	Long-term outcomes	Predictors of outcome following massive neonatal bowel resection
[26] Puoti & Köglmeier, 2023	Review	Children with intestinal failure due to SBS	Nutritional management	Contemporary nutritional management of pediatric SBS-associated intestinal failure
[27] Chandra & Kesavan, 2018	Review	Children with SBS	Multidisciplinary management	Contemporary nutritional, medical, and surgical treatment paradigms
[28] Belza et al., 2019	Clinical study	Children with SBS-associated intestinal failure	Prediction of enteral autonomy	Development of a disease-severity model for predicting enteral autonomy
[29] Muto et al., 2022	Review	Pediatric SBS	Multidisciplinary management	Overview of contemporary medical, nutritional, and surgical management
[30] Piper et al., 2020	Interventional study	Children with SBS	Microbiome modulation	Effects of *Lactobacillus* supplementation on intestinal microbiota
[31] Höllwarth & Solari, 2021	Review	Children with SBS	Nutritional and pharmacological management	Nutritional and pharmacological strategies supporting intestinal rehabilitation
